# Advances in Imaging in Evaluating the Efficacy of Neoadjuvant Chemotherapy for Breast Cancer

**DOI:** 10.3389/fonc.2022.816297

**Published:** 2022-05-20

**Authors:** Xianshu Kong, Qian Zhang, Xuemei Wu, Tianning Zou, Jiajun Duan, Shujie Song, Jianyun Nie, Chu Tao, Mi Tang, Maohua Wang, Jieya Zou, Yu Xie, Zhenhui Li, Zhen Li

**Affiliations:** ^1^Third Department of the Breast Surgery, The Third Affiliated Hospital of Kunming Medical University, Yunnan Cancer Hospital, Yunnan Cancer Center, Kunming, China; ^2^Department of Pathology, The Third Affiliated Hospital of Kunming Medical University, Yunnan Cancer Hospital, Yunnan Cancer Center, Kunming, China; ^3^First Department of the Breast Surgery, The Third Affiliated Hospital of Kunming Medical University, Yunnan Cancer Hospital, Yunnan Cancer Center, Kunming, China; ^4^Department of Radiology, The Third Affiliated Hospital of Kunming Medical University, Yunnan Cancer Hospital, Yunnan Cancer Center, Kunming, China

**Keywords:** neoadjuvant chemotherapy (NAC), breast cancer, evaluations of response, imaging, PCR

## Abstract

Neoadjuvant chemotherapy (NAC) is increasingly widely used in breast cancer treatment, and accurate evaluation of its response provides essential information for treatment and prognosis. Thus, the imaging tools used to quantify the disease response are critical in evaluating and managing patients treated with NAC. We discussed the recent progress, advantages, and disadvantages of common imaging methods in assessing the efficacy of NAC for breast cancer.

## 1 Introduction

The World Health Organization International Agency for Research on Cancer (IARC) released the world’s latest cancer burden data in 2020. New breast cancer cases reached 2.26 million in 2020, replacing lung cancer as the world’s most extensive cancer. In 2020, the number of new breast cancer cases in China was about 420,000, and the death toll reached 120,000 (1), placing a heavy burden on society. Therefore, research on the diagnosis and treatment of breast cancer has significant value.

In clinical practice, early breast cancer lesions can be directly treated by surgical resection, but for breast cancer with large primary foci or early metastasis, direct surgical resection cannot achieve the best therapeutic effect. NAC, one of the standard treatments for most breast cancers, refers to a systemic chemotherapy administered prior to the local treatment modality for primary tumors. It can lower the clinical stages of tumors, to facilitate breast conservation and render inoperable tumors operable (2). In recent years, NAC has attracted extensive attention. Although patients with breast cancer respond to NAC, significant differences exist. For instance, patients at the same stage and with the same molecular typing may show different responses to the same NAC. Study (3) shows that 10%~35% of patients are still insensitive to NAC, and disease progression can occur during treatment. Therefore, it is of great importance to timely and accurately evaluate the efficacy of NAC for breast cancer. During NAC, early evaluation of its efficacy is helpful for the clinical assessment of patients’ sensitivity to chemotherapy drugs, to guide subsequent precise drug use (4). In addition, NAC can reduce the burden of the primary tumor and achieve pathologic complete response (pCR) of axillary lymph node metastasis in more than half of patients (5). Thus, the possibility of axillary preservation is improved, and problems, such as upper limb edema, pain, and limited shoulder joint movement caused by the axillary lymph node dissection, are avoided (6). Therefore, accurate evaluation of the efficacy of NAC is critical to achieving individualized treatment of breast cancer.

**Figure 1:**
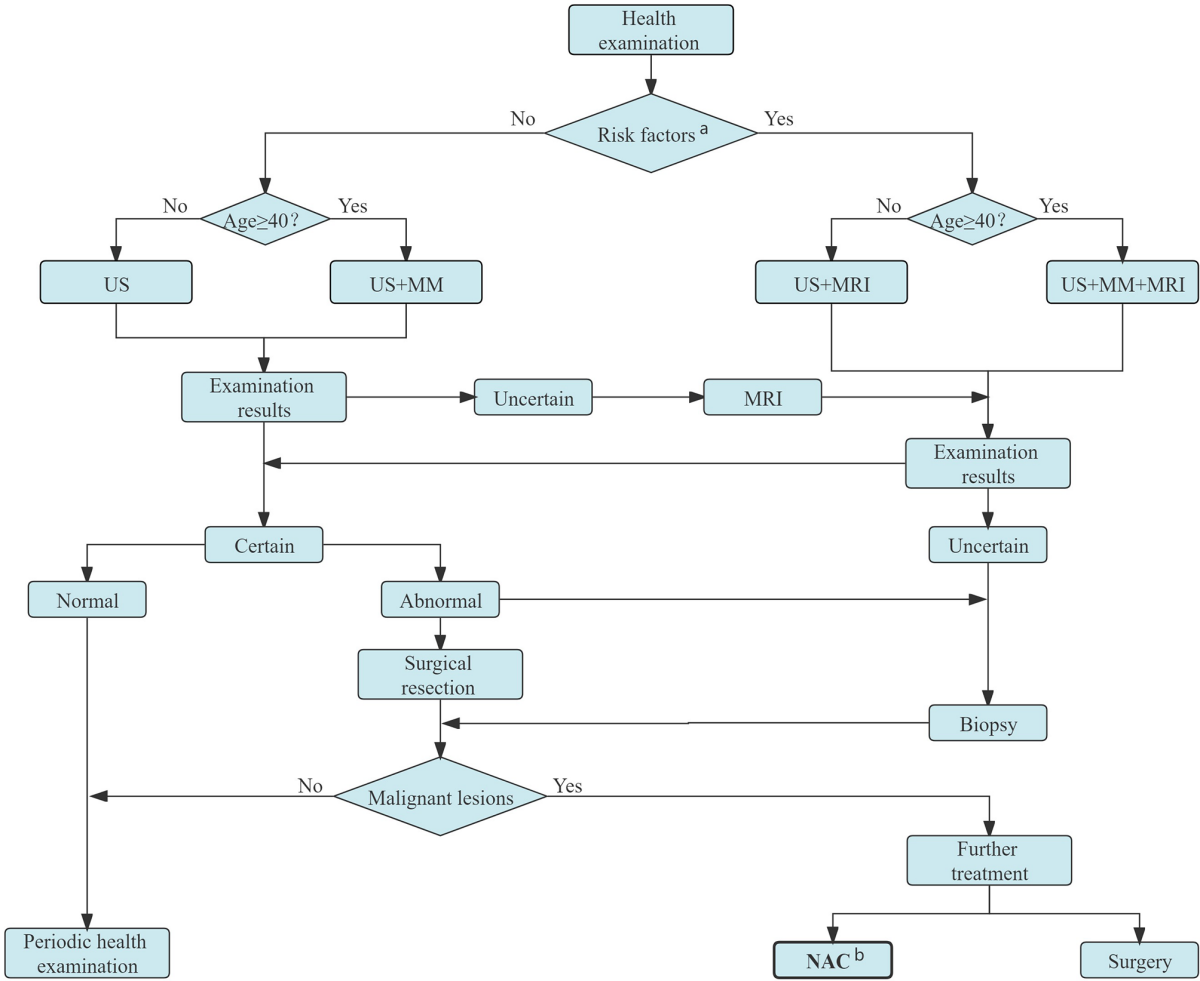
Candidates for NAC screening process in Yunnan Cancer Hospital. ^a^ Persons with obvious genetic tendency of breast cancer, history of LCIS or ductal or lobular dysplasia, or who experienced chest radiation before age 30. ^b^ Candidates for NAC: patients with inoperable breast cancer (IBC, bulky or matted cN2 axillary nodes, cN3 nodal disease, cT4 tumors), NAC is preferred for those with inoperable breast cancer (HER2-positive disease and TNBC if cT≥2 or cN≥1, large primary tumor relative to breast size in a patient who desires breast conservation, cN+ disease is likely to become cN0 with preoperative systemic therapy) and patients in whom definitive surgery may be delayed. US, ultrasound; MM, mammography; MRI, magnetic resonance imaging; NAC, neoadjuvant chemotherapy; LCIS, lobular carcinoma in situ; IBC, inflammatory breast cancer; HER2, human epidermal growth factor receptor 2.

The first stage of NAC process is patient selection. Ideally, not all the patients requiring adjuvant chemotherapy should receive NAC. The American Society of Clinical Oncology (ASCO), National Comprehensive Cancer Network (NCCN), Chinese Society of Clinical Oncology (CSCO), and other guidelines have recommended the selection of an intention-to-treat population. “Based on the actual clinical needs, and guided by the therapeutic purpose” is an important clinical practice NAC candidate selection principle (7). **Figure 1** shows the specific screening process of NAC candidates in Yunnan Cancer Hospital.

On the entry of the candidates into the NAC process, the NAC efficacy needs to be evaluated. The current methods used to assess the efficacy of NAC in breast cancer include clinical manifestations, laboratory examinations (8), imaging, pathology, and molecular examination (9). Current clinical examination mainly relies on doctors’ palpation to measure the size of the mass before and after NAC, to evaluate changes in the size. However, some problems may exist: subjective measurement and doctors’ evaluation inaccuracy, failure to differentiate tumor residue after chemotherapy from fibrosis or necrosis caused by chemotherapy, difficult perception of deeper and smaller lesions, and a high dependency on the doctors’ clinical experience.

As the gold standard for evaluating tumor response after chemotherapy, through pathological examination, changes, degeneration, necrosis, and tumor cell disappearance after chemotherapy, can be observed directly with high diagnostic accuracy. In particular, patients who achieved pCR after NAC have a better prognosis. A study (10) revealed that the degree of pathological reaction after NAC is closely related to the patients’ prognosis. Therefore, it is important to accurately evaluate and report pathological reactions after NAC. The WHO Classification of Breast Tumor Pathology and Genetics (2012 edition) lists eight assessment systems but does not explicitly recommend them (11). Currently, the commonly used pathological evaluation systems of NAC include Miller-Payne (MP) system, Residual Cancer Burden (RCB) system, Chevallier system, Sataloff system, and the AJCC ypTNM installment. Most of these evaluation systems classify post-chemotherapy reactions into pCR and non-pCR. Non-pCR patients are further categorize using different assessment systems by degree of response. The MP system is commonly used in the pathology departments in China (12), it compares the coarse needle biopsy specimen before chemotherapy with the surgical specimen after chemotherapy, and mainly evaluates the cell richness of residual tumor (which is divided into five grades) after NAC. However, as an invasive examination, pathologic examination is not actively applied in the treatment process. It must be performed after surgery; thus, the outcome of the efficacy evaluation is obtained late, and the sensitivity of the tumor to chemotherapy cannot be timely assessed. Therefore, it is difficult to adjust the treatment schedule in time, resulting in the best time for adjustment easily missed.

**Figure 2:**
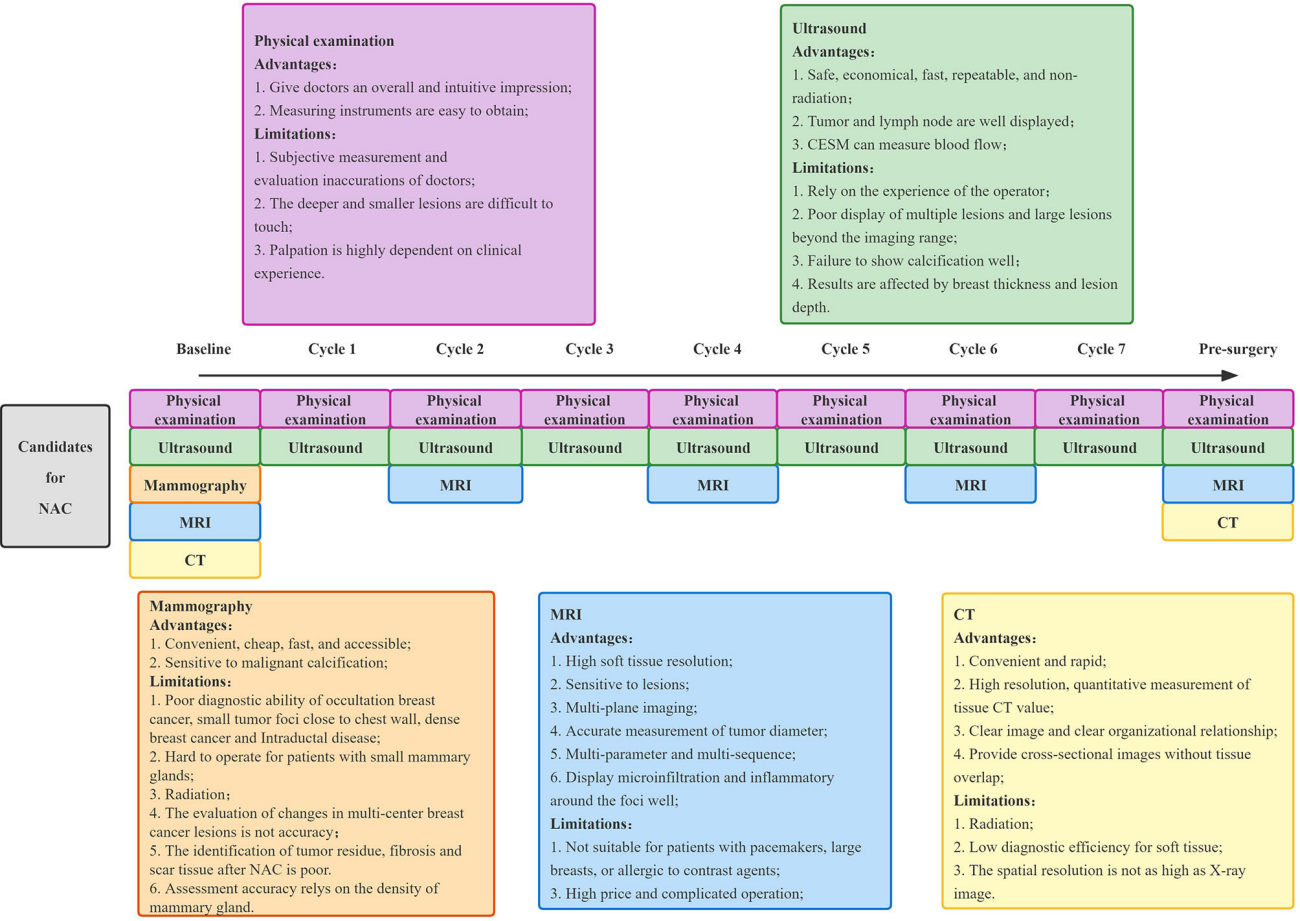
The model of imaging technology to assess the efficacy of NAC for breast cancer in Yunnan Cancer Hospital. NAC, neoadjuvant chemotherapy; MRI, magnetic resonance imaging; CT, computed tomography.

Imaging, as one of the most important methods to evaluate the efficacy, has the advantage of being non-invasive and can be used throughout the whole process of breast cancer treatment, including a pre-treatment baseline image to determine the scope of the lesion, treatment efficacy evaluation during NAC, and post-treatment residual lesion evaluation. Imaging examination can not only objectively be used to evaluate the efficacy of NAC, but also provides an important basis for clinicians to choose an appropriate surgical approach and determine patients’ prognosis. At present, the commonly used clinical imaging evaluation methods include mammography, ultrasound, magnetic resonance imaging (MRI), and positron emission tomography CT (PET-CT). **Figure 2** shows the imaging evaluation process of NAC efficacy for breast cancer in Yunnan Cancer Hospital.

Nevertheless, there is no unified guideline for the imaging evaluation of NAC response, and in recent times, the efficacy evaluation is mainly based on changes in tumor size, changes in the degree of ultrasound or MRI enhancement, and the form of tumor regression. Currently, the Response Evaluation Criteria in Solid Tumors (RECIST) 1.1 (13) remains the most used clinical evaluation criteria. This is done by measuring the change in the longest diameter of the lesion before and after NAC to evaluate efficacy, with a focus on the observance of change in the longest diameter of the lesion. For multifocal lesions, a comparison of the sum of the longest diameter measurements of all lesions should be included. Tumor remission after treatment is categorized as remission or no remission according to RECIST criteria. Remission included: 1) complete remission (CR) or no tumor residue; 2) partial response (PR), which was when the longest diameter of the tumor decreased by >30%. No remission included: 1) disease progression (PD), which was when the maximum diameter of the tumor increased by >20% or a new lesion appeared; 2) stable disease (SD), when the tumor size changes are between those of partial remission and progression. However, there are some limitations of RECIST 1.1. Tumor regression can be divided into centripetal (when the tumor size decreases significantly) and non-centripetal regression (when its size does not change significantly), RECIST 1.1 is not suitable for the efficacy evaluation of non-centripetal regression tumor. Moreover, RECIST 1.1 is far from being adequate for evaluating NAC efficacy of breast cancer based on tumor diameter only. There is still no guideline or standard to guide the selection of important evaluation indicators such as functional magnetic resonance and three-dimensional US, which needs to be further improved.

There are different imaging methods suitable for evaluating NAC efficacy in different stages of breast cancer, and each imaging method also has its own area of emphasis for evaluating efficacy in breast cancer of different molecular types. It is crucial for clinicians to familiarize themselves with the progress, advantages, and disadvantages of these imaging methods in evaluating NAC efficacy. Currently, several studies, reviews, and meta-analyses exist on imaging assessment of NAC. To this end, this article reviews the value and recent progress of imaging in evaluating NAC efficacy for breast cancer based on the study of a large number of relevant literature.

## 2 Evaluation of the Efficacy of Mammography on NAC in Breast Cancer

### 2.1 Mammography

Mammography evaluation shows signs of tumor lesion calcification disappearance and burr shortening or disappearance after breast cancer NAC. However, the above features have low accuracy in evaluating the efficacy of NAC. The evaluation of efficacy after NAC by mammography is mainly based on changes in tumor size and density. Two retrospective studies (6, 14) showed poor consistency between mammography measurement and pathological results after NAC, with a moderate level of consistency correlation coefficient (CCC) at only 0.52-0.58. Therefore, most experts consider mammography to be unsuitable for the evaluation of NAC efficacy. In addition, a recent prospective study (15) compared the size of tumors evaluated by mammography, ultrasound, and tomosynthesis after NAC, and reported the sensitivity, specificity, positive predictive value (PPV), and negative predictive value (NPV) of mammography as 0.65, 0.81, 0.52, and 0.88, respectively. The agreement rate between mammography and pathological assessment in pCR was only 43%. Thus, although mammography is highly specific in detecting tumors, it misestimates the tumor size in about half of patients.

Although mammography can describe malignant calcification well, microcalcification is not reliable evidence of the persistence of residual tumors. A previous study (16) shows that residual microcalcification after NAC is not always related to a residual tumor burden. Residual microcalcification can represent both the residual tumor and necrotic tumor cell products after treatment. When calcification persists after NAC, compared with mammography, the size on MRI is more consistent with the pathological results (17). Feliciano et al. (18) suggested that, although not all residual microcalcification on mammography after NAC reflect residual tumor and 44.8% of residual microcalcification is unrelated to the residual tumor, all microcalcification in the tumor should be completely excised.

In conclusion, mammography has certain limitations in assessing the efficacy of NAC in breast cancer: 1) it is unable to accurately determine the changes of multicenter breast cancer lesions; 2) it has X-ray radiation and cannot be used to examine frequently; 3) it is not suitable for the identification of tumor residue, fibrosis, and scar tissue after NAC; and 4) residual microcalcification after NAC is often overestimated. Therefore, The American College of Radiology recommended mammography, ultrasound, and MRI as the highest grade (grade 9) at baseline (pre-NAC), while MRI was still recommended at grade 9 during and after treatment; however, ultrasound and mammography were reduced to grade 8 and grade 7, respectively (19).

### 2.2 Contrast-Enhanced Spectral Mammography (CESM)

CESM, an examination combined with contrast agents based on conventional mammography, is a new mammary gland imaging technique used to obtain low energy and subtraction images after post-processing them through rapid high and low energy dual exposure, after intravenous injection of contrast agents. It can show abnormal vascular proliferation in tumor tissues, thus significantly reducing the false positive and false negative rates and improves the sensitivity and accuracy of detection (20, 21).

MRI is currently the most recommended imaging for efficacy assessment during NAC. One study (22) compared the performance of CESM and MRI in evaluating the tumor response to NAC treatment at different stages and showed different consistency of CESM and MRI in measuring the size of lesions at different treatment stages. The consistency of the measurement of the lesion size before, during, and after NAC was 0.96, 0.94, and 0.76, respectively, and both of CESM and MRI were prone to underestimating the residual lesions. However, in another retrospective study, Patel et al. (23) compared the mean residual tumor size measured by CESM and MRI in 65 patients on NAC, using surgical pathology results as a reference standard. The residual lesion size measured by CESM and MRI was found to correlate well with the pathology results (r of 0.77 and 0.80, respectively), and the mean residual lesion measured on both was -1~1 cm different from the pathological results. Similarly, Barra et al. (24) also proved that CESM can be used to evaluate residual tumor size after NAC, with good correlation and consistency with pathological results. A previous prospective study involving 21 breast cancer patients (25) evaluated CESM in predicting tumor response to NAC; the specificity, sensitivity, NPV, and PPV of 91%, 40%, 80%, and 62.5%, respectively, show good efficacy of CESM in predicting tumor response after NAC. However, the sample size in this study is relatively small, and further large-sample studies are needed to confirm the reasons for the low CESM sensitivity. CESM also performed well in predicting pCR early after NAC. Xing et al. (26) retrospectively quantified the enhancement intensity of CESM in 111 patients by calculating the percentage of grey value reduction percentages (ΔCGV). The results showed statistically significant differences in ΔCGV between the pCR and non-pCR groups, indicating that ΔCGV obtained based on CESM images can be used as a quantitative indicator for early prediction of pCR after NAC.

The results of previous studies suggest that CESM can be used to assess the efficacy of NAC and has good application in predicting pCR early after NAC. Due to the shorter examination time of CESM, better patient tolerance, and lower price, CESM has a broader prospect in the evaluation of the pathological response of breast cancer to NAC. However, the technique requires multiple breast images in different positions after contrast injection; hence, its use is limited in patients with contrast agent allergies. In addition, more extensive studies are necessary to better understand the efficacy evaluation of NAC for different molecular subtypes of breast cancer, exploration of tumor regression patterns, assessment of efficacy after NAC for tumors containing calcified foci, and assessment of CESM radiomics.

## 3 Evaluation of the Efficacy of Ultrasound on NAC in Breast Cancer

Ultrasound is a safe, fast, reproducible, and economical imaging evaluation method. Conventional ultrasound can describe the size, morphology, and boundary of tumors. Ultrasound imaging technology can further evaluate the tumor volume, internal blood vessels, and other subtle structures, as well as the softness and hardness of the tumor (27). The China Anti-Cancer Association Breast Cancer Guidelines strongly recommend that ultrasound be used regularly to reassess the tumor’s treatment response after every two NAC cycles (28). Therefore, ultrasonography has a place in the evaluation of the efficacy of neoadjuvant therapy. Because ultrasound is reproducible, cheap, and non-invasive, it is now more widely used in China.

### 3.1 Ultrasound

#### 3.1.1 Two-Dimensional Ultrasound, Color Doppler Ultrasound

Two-dimensional ultrasound can reflect the size, morphology, boundary, and other information of breast lesions and show the structure and morphology of lymph nodes well (27). However, it is greatly influenced by the operating doctor and cannot accurately distinguish between tumor and normal gland tissue. Hence, the two-dimensional ultrasound is unable to accurately monitoring the size change in response to NAC, and its specificity in assessing the NAC response is low. It is not recommended for NAC efficacy evaluation.

**Figure 3:**
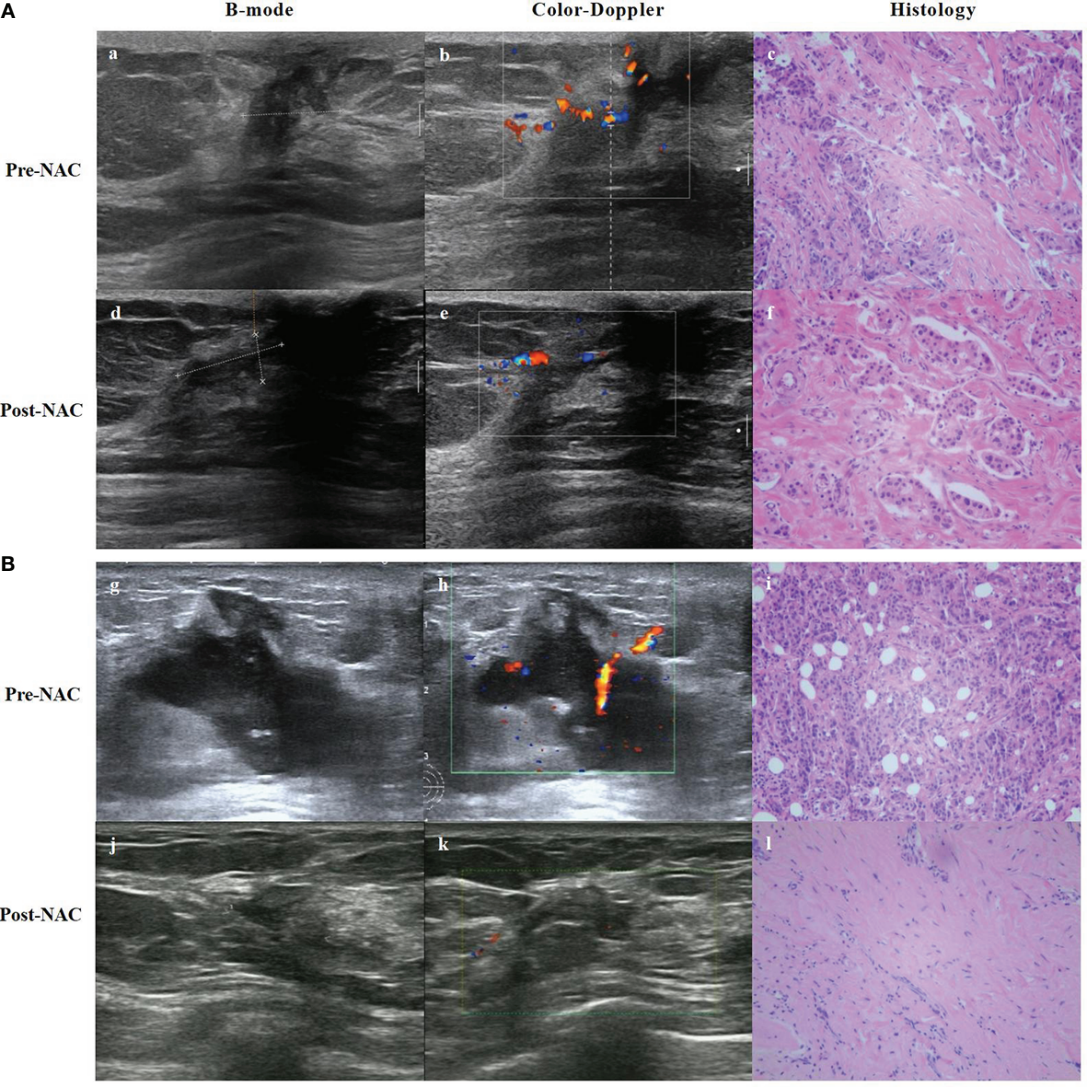
B-mode, Color-Doppler ultrasound and histology from a partial responder **(A)** and complete responder **(B)** before start of NAC (Pre-) and after 8 course of NAC (Post-). **(A)** (a) B-mode ultrasound: the tumor was hypoechoic. (b) Color-Doppler ultrasound: moderate peripheral vascular signals. (c) Microscopic image of core-needle biopsy. After 8 course of NAC. (d) B-mode ultrasound: the echogenicity increased. (e) Color-Doppler ultrasound: less residual vascularization compared to baseline. (f) Microscopic image after NAC shows residual tumor cells, but reduced compared to baseline. **(B)** (g) B-mode ultrasound: the tumor was hypoechoic. (h) Color-Doppler ultrasound: moderate intralesional and perilesional vascularization. (i) Microscopic image of core-needle biopsy. After 8 course of NAC. (j) B-mode ultrasound: the echogenicity increased and tumor volume decreased. (k) Color-Doppler ultrasound: almost no vascular spots. (l) Microscopic image after NAC presents visible stromal tissue, no visible tumor cells.

Tumor vascular distribution is an alternative indicator of tumor burden. In addition to evaluating tumor size and morphology, color Doppler ultrasound can also be used to evaluate tumor vascular distribution through a variety of parameters that compare tumor changes before and after chemotherapy, to assess the response to chemotherapy (**Figure 3**). Chemotherapeutic drugs can destroy the neovascularization of tumors through the blood circulation, thereby reducing the pressure of tumors on the surrounding tissues, and hence, the hemodynamic changes can be used as an indicator to assess the efficacy of treatment (29). A study (30) using color Doppler ultrasound measured the sizes of tumors after NAC and compared them to histopathological results. The study found that the sensitivity, specificity, PPV, and NPV were 91.7%, 38.5%, 57.9%, and 83.3%, respectively. It showed that Doppler ultrasound has high sensitivity and can accurately reflect the efficacy of NAC in breast cancer. In recent years, with the development of color Doppler ultrasound technology and the improvement of diagnostic technology, it has become one of the most widely used methods to evaluate NAC efficacy. However, when chemotherapeutic drugs act on the tumor vasculature and inflammatory changes occur in the surrounding tissues, the vasculature may become narrowed and occluded, and in this case, the measurement results will be affected. Therefore, the application of color Doppler is somewhat limited.

#### 3.1.2 Contrast-Enhanced Ultrasound Technology (CEUS)

CEUS, a purely blood pool imaging technique, detects micro-vessels to show the neovascularization of breast tumors and the perfusion pattern of blood flow, to obtain contrast-enhanced images. It shows the morphology and distribution of lesions and blood vessels clearly (29). CEUS can be used to evaluate the mode of lesion enhancement, and to quantitatively evaluate some indicators by generating time-intensity curves, such as rise time, mean passage time, time to peak, peak intensity, and area under the curve (AUC). Although it is most intuitive and straight forward to assess the efficacy of breast cancer after NAC by measuring changes in the size of the lesions, there are limits in measurements, using this method; this is because of the operator’s subjective assessment and because the masses do not all show centripetal retractions after NAC. Thus, the relative change rate of the size of contrast parameters is of important clinical significance.

Changes in tumor blood vessels after NAC precede morphological changes, so the difference in blood perfusion is critical for NAC efficacy evaluation (29). Especially for localized liquefaction necrosis of the tumor, CEUS has a higher accuracy in assessing the mass size compared to a two-dimensional ultrasound. A study (31) has shown that CEUS can be used to assess the clinical response of tumors to NAC, and the sensitivity and specificity of predicting pCR after NAC were 95.7% and 77.5%, respectively. Other studies concluded that CEUS is similar to MRI in predicting pCR and has a higher correlation with pathological examination in evaluating the size of residual lesions, even higher than that of MRI (32, 33). Huang et al. (34) also identified CEUS as a potential tool for predicting NAC response in locally advanced breast cancer patients. Compared with other molecular subtypes, triple-negative and HER2+/ER- subtypes responded better to NAC. Notably, breast cancer is highly heterogeneous, and the same NAC regimen may produce different responses for different molecular subtypes. Therefore, it is urgent to study the correlation between clinical/biological indicators and CEUS parameters.

In summary, CEUS, as a cutting-edge research field, has good clinical application in assessing the efficacy of NAC for breast cancer patients who cannot undergo dynamic contrast-enhanced MRI (DCE-MRI) or require multiple evaluations; it can measure the maximum diameter of lesions after NAC more accurately than conventional ultrasound and is in good agreement with histopathological results. However, large sample multi-center studies are needed to further explore more sensitive indicators of NAC response. The limitation of CEUS in clinical practice is the poor visualization of multiple lesions and large lesions beyond the imaging range.

#### 3.1.3 Automated Breast Volume Scanner (ABVS)

ABVS uses the advantages of multi-plane remodeling to create a three-dimensional ultrasound imaging of the breast tissue. It can better reflect the growth mode of breast tumors and the relationship with the surrounding tissues through automatic, full-volume, and coronal scanning of the breast (35). Since it is automatically scanned and digitally stored, it relies less on physician operations compared to traditional ultrasound, and the examined images can be reviewed (36). Using the ABVS, multiple masses can also be examined at once and shown in the same view, overcoming the limitations of conventional ultrasound (35). At present, the superiority of ABVS in identifying benign and malignant breast tumors has been recognized (37). Further studies explored its utility in predicting the efficacy of patients with NAC to provide a better basis for further clinical diagnosis and treatment.

A Chinese study (38) first explored the use of ABVS to predict pathological outcomes after four cycles of NAC by assessing the proportional changes in primary tumors measured after two NAC cycles. The results suggest ABVS as a valuable tool for the early assessment of pCR after NAC. However, it is less reliable in predicting adverse pathological outcomes (Miller-Payne grades 1 to 3). Another study (39) compared the efficacy of ABVS and MRI in assessing tumor response; the two had a reasonable correlation for differences in the longest tumor diameter measurements (CCC 0.73). Regardless, ABVS has higher patient satisfaction, indicating it can effectively be used to monitor patients during NAC. However, Park et al. (40) compared the accuracy of mammography, digital breast tomography (DBT), ABVS, and MRI in assessing the degree of tumor residual after NAC. The results showed that ABVS had the lowest reliability in predicting residual tumor size and pCR and tended to underestimate residual tumors. This suggests that ABVS may not be sensitive enough to distinguish chemotherapy-induced fibrosis and hypoechoic tumors after NAC. The differences in the above trial results may be due to tumor heterogeneity, variability of pathological size assessment, or differences in study design; moreover, retrospective studies may lead to bias due to incomplete data. Therefore, we must interpret these results rationally. More prospective studies and larger case series are required to explore ABVS in assessing the efficacy of tumor NAC.

### 3.2 Ultrasound Elastography

The tumor tissue changes complicatedly during the treatment, including cell degeneration, necrosis, liquefaction, slow proliferation rate, tissue fibrosis, and focal tissue hardness. Pathological biopsy after NAC showed that patients with ineffective (or effective) treatment had higher (or lower) cancer cell density, resulting in changes in the elastic coefficient before and after NAC. Therefore, ultrasound elastography can be used to evaluate the efficacy of NAC (41). In recent years, ultrasound elastography has been widely used in the evaluation of NAC, while strain elastography (SE) and shear wave elastography (SWE) are commonly used for breast cancer. SE enables qualitative and quantitative analyses of tissue softness and hardness to evaluate NAC efficacy, by comparing the elastic score and strain rate ratio before and after NAC. SWE reflects the efficacy of NAC for breast cancer by measuring the value of tissue elasticity, that is, the absolute value of Young’s modulus (42).

Studies in other countries (43, 44) found that the sensitivity and specificity of assessing tumor changes by SE after two treatment cycles were 83.3% – 84% and 80% – 85%, respectively. It is shown that SE can predict the NAC response of locally advanced breast cancer within two weeks of treatment with high sensitivity and specificity. Furthermore, the elastic changes in the tumor response to NAC can be used as an early response marker in the treatment process. A prospective study by Jing et al. (45) used SWE for the first time to predict the response of breast cancer patients to NAC. The relative change of tumor stiffness after two NAC cycles was significantly associated with the pathological response of postoperative specimens, with sensitivity and specificity of 72.9% and 85.7%, respectively. This indicates that the change in tumor stiffness is a handy predictive parameter for judging the efficacy of NAC for breast cancer; thus, SWE can be used as an effective method to guide NAC. Lee et al. (46) confirmed that the diagnostic efficacy of ultrasound combined with SWE for NAC was almost similar to that of MRI (P>0.05), and the elastic value of the residual tumor tissue after NAC was up to a maximum of 116 ± 74.1 kPa, which is much higher than that of non-residual tumor tissue (26.4 ± 21.0 kPa). Ma et al. (42) compared the diagnostic performance of SE and SWE in predicting the NAC response in breast cancer; the results showed similar diagnostic performance in the early prediction of NAC response. Regardless, SWE is superior to SE in the early prediction of NAC resistance. Ultrasound elastography also has certain value in predicting pCR of tumors. A comparative study comparing SWE and MRI (47) showed that the ability of pCR prediction (when the reduction in the average lesion hardness was combined with tumor diameter on conventional ultrasound) was close to that of MRI, with AUC of 0.92 and 0.96, respectively. However, there is a need for further studies on the combination of elastography and other evaluation methods, and its detection efficiency in tumors of different phenotypes (48). Although elastography technology has high diagnostic efficiency in assessing the efficacy of NAC, there are no reports of changes in breast cancer treatment strategies based on elastography evaluation results.

One study showed breast thickness and lesion depth as important factors affecting the quality of elastography images (41). In addition, the uneven internal hardness (caused by the liquefaction and necrosis of the mass) and higher hardness (caused by fibrosis or hyaline degeneration) of the original lesions after NAC can affect the measurement results. The operator’s experience and knowledge also have a significant influence on the measurement results. Sufficient compression and precise positioning of the tumor region must be ensured (44). Therefore, the application has some limitations, and further improvements are needed in the future. In addition, results from the evaluation of elastography compared to other imaging modalities are lacking.

### 3.3 Quantitative Ultrasound (QUS) and Diffused Optical Tomography With Ultrasound (OPTI-MUS)

QUS utilizes changes in the acoustic properties of tissues to reflect changes in their microstructure. It works by scanning the breast tumors using a clinical ultrasound system; then, the ultrasound radio frequency (RF) data within the tumor regions of interest were retained and displayed as a frequency spectrum using a fast Fourier transform (FFT). The analysis of the power spectrum leads to various features like spectral slope (SS), spectral intercept (SI) at 0 MHz, mid-band fit (MBF), average scatterer diameter (ASD), average acoustic concentration (AAC), attenuation coefficient estimate (ACE), and spacing among scatterers (SAS) (49). Its parameters reflect both the elastic and microstructural properties of the tissue. Its simple operation, low cost, and non-requirement of an exogenous contrast agent gave the technique partial attention. In a preliminary clinical study (50), two parameters of QUS were used to determine the pathological response of patients with locally advanced breast cancer after NAC treatment. The sensitivity and specificity in the first and fourth cycles were 77% vs. 86%, and 83% vs. 100%, respectively. Thus, it can be used for early detection of tumor response to NAC. Sannachi et al. (49) used a combination of QUS parameters, texture, and molecular characteristics to monitor the response to NAC treatment. In the first, fourth, and eighth week after treatment, the accuracy of this combination for predicting treatment response was 78%, 86%, and 83%, respectively. However, the accuracy of QUS parameter prediction, only, at these three-time points is less than 60%. There are few studies on QUS predicting breast tumor response after NAC treatment, and the existing research are insufficient. Based on current preliminary studies on the objective results, more extensive prospective studies are necessary to clarify the evaluation effectiveness of QUS in NAC.

OPTI-MUS is a new imaging technology that combines conventional ultrasound and diffused optical tomography through specific technological means. Diffused optical tomography uses the diffuse scattering effect of tissue on the multi-wavelength laser to complete the three-dimensional imaging of tissue physiological information. Measuring the total hemoglobin (HBT), deoxyhemoglobin (HBO2), and other parameters in each section of the tumor region indirectly reflects the tumor angiogenesis activity to evaluate the efficacy of NAC at the molecular level (51). OPTI-MUS is associated with NAC response (52–54). Tran et al. (55) obtained ultrasound and OPTI-MUS data related to the start of NAC at 0, 1, 4, and 8 weeks, and before surgery, respectively. The results showed that individual QUS and OPTI-MUS parameters, including the SI, HBO2, and HBT were significant markers for response after one week of treatment (p < 0.01). Multivariate combinations increased the sensitivity, specificity, and AUC. QUS and OPTI-MUS are both non-invasive and relatively economical, rapid examinations. However, challenges, such as errors in the diagnosis of small and superficial tumors, persist; thus, its application in monitoring the efficacy of NAC in combination with other imaging examinations should be further researched.

## 4 Evaluation of the Efficacy of MRI on NAC in Breast Cancer

There are various diagnostic modalities to assess the efficacy of breast cancer after NAC. Although many studies have tried to determine the best imaging method in evaluating the efficacy of NAC, no consensus has been reached. To date, MRI is the most used accurate imaging method to assess the extent of tumor residual after NAC (56). Moreover, breast MRI multiparametric imaging can quantify and visualize multiple functional processes simultaneously at the cellular and molecular levels. This clarifies the therapeutic response of breast cancer and assesses the response efficacy of NAC earlier, for timely clinical adjustment of treatment regimens.

### 4.1 The Conventional MRI

MRI has high soft-tissue resolution and can effectively distinguish residual tumors from post-chemotherapy fibrotic or necrotic tissue. The therapeutic effect can be judged mainly by morphology and by measuring the change in the maximum diameter of the lesion. Therefore, to some extent, MRI can reflect the actual size of the mass. The length and diameter measurements were also based on RECIST 1.1 efficacy assessment criteria, and tumor responses were classified as either responsive (CR and PR) or non-responsive (SD and PD). The presence or absence of residual lesions after NAC of breast cancer, accurate size measurement, and accurate pCR prediction directly affect the adjustment of treatment plan and the choice of surgical approach in clinical practice. Compared with mammography, ultrasound, or clinical palpation, lesion size measured by MRI has a higher correlation with pathological examination. A prospective ultrasound trial (57) enrolled 174 patients with invasive breast cancer who were treated with NAC. Preoperative measurements of all lesions were assessed by mammography, clinical examination, and MRI, to detect the correlation between the accuracy of pathologic CR and final pathologic size. Ultimately, they found that clinical examination often underestimated residual tumor size. In contrast, mammography tended to overestimate, and MRI appeared to reflect the size of residual lesions more accurately, consistent with previous results. Therefore, MRI is still the most accurate method to measure the maximum diameter of NAC when considering the efficacy assessment after NAC only. The accuracy of MRI measurement of residual lesions in different molecular subtypes of breast cancer is, in that order, best in triple negative and HER2 over-expression (58, 59), while underestimation of lesions is common in the Luminal type (58–60). The PPV and NPV for predicting pCR were both highest in triple-negative breast cancers, while PPV in HER2 over-expressed breast cancers was second only to triple-negative breast cancers.

Post-NAC MRI shows two main types of tumor shrinkage: concentric and nested or dendritic shrinkage (61). It can accurately evaluate concentric shrinkage, but the conventional MRI has limited value in assessing tumors with nested or dendritic shrinkage. It is split into many small pieces and pathologically shows multicentric and discontinuous residual tumors (62).

### 4.2 Dynamic Contrast-Enhanced MRI (DCE-MRI)

DCE-MRI is highly sensitive to changes in tumor presence and angiogenesis. It is most used for semi-quantitative analysis parameters to assess NAC efficacy in breast cancer, including the early intensification rate, time to peak, maximum intensification rate, and apparent diffusion coefficient, reflecting tissue vascular density and vascular permeability. It has further been demonstrated that some quantitative parameters, such as volume transfer constant (K^trans^), rate constant (K_ep_), and extracellular space volume ratio (V_e_), can be used for early prediction of breast cancer response to NAC (35). DCE-MRI curve changes can also be used to evaluate the efficacy of NAC for breast cancer. Generally, time-signal intensity curve (TIC) morphology is divided into type I (slow and continuous enhancement type); II (platform type); and III (clearance type). When the curve shape changes from low to high grade (e.g. from type II to III) after treatment, it indicates that the tumor is more aggressive and chemotherapy is ineffective. On the contrary, when it decreases, it suggests that the treatment is effective. However, at present, there is no unified standard for the quantitative index and threshold value of using DCE-MRI to assess the efficacy of NAC.

The correlation between pathological tumor diameter after NAC and DCE-MRI tumor diameter was reported to be closer than that of palpation or ultrasound (63). Furthermore, tumors with nested or dendritic shrinkage after NAC can be evaluated for efficacy with DCE-MRI or quantitative diffusion-weighted MRI (DWI-MRI) (64, 65). A meta-analysis (66) that included 18 studies (969 breast cancer patients) showed that DCE-MRI has a combined sensitivity and specificity of 0.80 and 0.84, respectively. DCE-MRI has a higher sensitivity for early prediction of response to breast cancer, compared with assessment of tumor response after NAC completion. It is an effective method for the dynamic monitoring of NAC efficacy and can also predict the pCR response of breast cancer after NAC. DCE-MRI was recommended to evaluate the efficacy of NAC in the RECIST guidelines (35). Other studies (67, 68) showed that semi-quantitative and quantitative analyses based on DCE-MRI had certain value in early prediction of NAC efficacy. In a study on quantitative DCE-MRI assessment of NAC efficacy for breast cancer, Li et al. (69) noted that the changes in quantitative parameters, Ktrans and Kep, which reflect blood perfusion and infiltration, showed statistically significant differences between the pCR and non-pCR groups after two cycles of NAC; with subsequent similar conclusions in another study (70). In the early stages of NAC, the diagnostic efficacy of combining semi-quantitative and quantitative DCE-MRI parameters may be higher. Changes in the maximum tumor diameter in the advanced enhancement stage of DCE-MRI can be used to better evaluate the tumor’s sensitivity to chemotherapy drugs. When the maximum tumor diameter is reduced by < 25%, there is a high possibility of malignant tissue residual, while in patients with pCR monitored by DCE-MRI, the tumor diameter is reduced by > 45% (71). Therefore, during NAC treatment, changes in tumor diameter and Ktrans and Kep parameters in DCE-MRI images, can be used as imaging indicators to evaluate the degree of tumor remission, thus providing more useful information for the formulating surgical plans. Fukuda et al. (72) evaluated the extent of tumor remission in DCE-MRI after NAC by imaging and performed a consistency test between imaging diagnosis results and pathological findings. They reported an accuracy of up to 88.7%, with a higher accuracy of 93.2% and 90.9% for Luminal and triple negative breast cancer, respectively, and a lower accuracy of HER2 over-expression breast cancer.

Obviously, according to current data, pCR prediction by imaging does not yet meet clinical expectations, and patients are still not exempt from surgery by virtue of a negative DCE-MRI result. However, MRI is still the most accurate method to evaluate residual tumor and predict pCR among all imaging evaluation methods. Limitations in the use of DCE-MRI are the lack of standardization of the DCE protocol and the possible overestimation due to necrosis, inflammation, fibrosis, or scar tissue caused by chemotherapy. At the same time, the antivascular effect of certain chemotherapeutic drugs and the presence of ductal carcinoma *in situ* (DCIS) may be underestimated due to poor imaging (31). Factors such as high cost, use of contrast agents, and selectivity for patients further limit its use (73).

### 4.3 Diffusion-Weighted Imaging (DWI)

Although DCE-MRI is currently a reliable technique for assessing NAC response, there are still difficulties in using it to predict postoperative pCR (74). DWI is used to evaluate NAC efficacy by probing the diffusion capacity of water molecules in living tissues, i.e., measuring apparent diffusion coefficient (ADC) values and performing quantitative analysis. It is, thus sensitive to cell density, membrane integrity, and tissue microstructure (75). Therefore, DWI may provide complementary information for predicting chemotherapy response.

An increasing ADC values in the early stages of NAC in breast cancer is an important indicator to assess the final chemotherapy outcome of the tumor. After the second cycle of NAC, ADC values showed statistically significant differences between the pCR and non-pCR groups (76). According to Iwasa (77), the increasing tumor ADC values at the end of the first cycle of NAC was also closely related to the final pathological remission tumor degree, with an AUC of receiver operating characteristic (ROC) for predicting pCR of 0.9. It is suggested that DWI can be used to evaluate the efficacy after the first cycle of treatment, which may prolong the time to adjust clinical protocols. In 2018, a prospective multicenter trial in the ultrasound (75) recruited 138 breast cancer patients to determine whether changes in ADC could predict pCR after NAC. It reported that parameters of DWI were more predictive of post-NAC pCR after 12 weeks of treatment, relative to the baseline characteristics [AUC:0.72, 95% CI:0.61-0.83]. The same conclusion was reached in another study (78).

Changes in ADC values correlate with the molecular subtypes of breast cancer. Further studies by Richard (79) and Bufi (80) on different molecular subtypes of breast cancer suggest that ADC value could be used as a predictor of efficacy before NAC in triple-negative type and over-expressed HER2 type breast cancer. However, in Luminal type breast cancer, there was no significant difference in tumor ADC value before NAC among different pathological response groups. Liu et al. (81) analyzed the ADC values of 176 patients with different molecular subtypes of breast cancer before and after NAC, and found that only the triple-negative breast cancer had significant difference in ADC values between the pCR and non-pCR groups before NAC; whereas, other molecular subtypes had no significant difference. There were significant differences in ADC values between pCR and non-pCR groups in each subtype of breast cancer after NAC. This conclusion indicates that due to the existence of multiple subtypes of breast cancer, the final efficacy evaluated by ADC value before NAC is limited to triple negative breast cancer and HER2 over-expressed breast cancer (82).

Although studies have shown that DWI can predict NAC response, its limitations include high sensitivity to movement, and thus, it is subject to motion artifacts due to respiratory and cardiac motions, poor spatial resolution, and difficulty in assessing certain breast cancer subtypes, such as invasive lobular carcinoma (83). Therefore, in the evaluation of residual tumors, it should not just be used as a single indicator to assess whether the tumor has achieved CR, if possible; but to combine multiple indicators such as tumor diameter reduction and increasing ADC value, for a comprehensive assessment of the tumor.

#### 4.3.1 Intravoxel Incoherent Motion Imaging (IVIM)

IVIM is a new DWI-based technique that separates the micro-perfusion effect of capillaries in tissues from the diffusion effect of water molecules to obtain the diffusion coefficient of water molecules alone (D), the pseudo-diffusion coefficient due to microcirculatory diffusion (D*), and the perfusion fraction (f), which may have a good potential for predicting NAC effects. Several studies have confirmed the potential value of the IVIM model in monitoring chemotherapy response in a variety of malignancies, such as liver cancer (84), head and neck tumors (85), and nasopharyngeal carcinoma (86); however, its studies on the efficacy of NAC in breast cancer are less available. Studies (87, 88) concluded that the parameters of IVIM had a good predictive performance for the pathological response. They observed that patients with higher baseline f values, higher on-treatment D values, and lower on-treatment f values responded better to NAC. Patients in the pCR group showed more significant changes in D and f values than in the non-pCR group. Changes in D values after two cycles of NAC treatment had a good predictive performance for differentiating between pCR and non-pCR. Another study (89) found no significant changes in D* and f values before and after NAC and concluded that they did not predict tumor response. In conclusion, more studies are needed to explore IVIM in assessing the response to NAC. In addition, molecular subtypes of breast cancer are associated with different IVIM parameters. Kim et al. (90) found that low tissue diffusion was primarily detected in tumors with high Ki-67 and Luminal B.

IVIM model has the possibility of increasing ADC value to predict NAC efficacy. However, few studies exist on the application of IVIM model in the efficacy evaluation and prediction of NAC in breast cancer, and further research and confirmation are still needed. IVIM parameters are affected by many factors including respiration collection method, fitting method, and tumor heterogeneity (91–93), resulting in poor repeatability.

#### 4.3.2 Diffusion Tensor Imaging (DTI)

DTI is considered an extension of DWI, which characterizes water motion by measuring it in six or more directions. DTI quantifies two parameters: mean diffusion coefficient (MD) and fractional anisotropy (FA). MD is an estimate of mean anisotropy, and FA reflects the degree of anisotropy (94). Although the early percentage change in tumor FA correlated weakly with pCR, the significant correlation with pathologic tumor volume suggests that this metric warrants further evaluation (95). Furman et al. (96) demonstrated the ability of DTI to monitor breast cancer response to NAC. It found that DTI monitors changes in diffusion tensor parameters during NAC with similar efficiency to DCE; the final pathological assessment had good agreement. Moreover, DTI provided an accurate percentage change in size when measuring changes in tumor volume rather than estimating within a wide range. Currently, DCE is the primary MRI method for assessing breast cancer response to NAC. However, DTI has significant advantages over DCE, such as no contrast injection and relatively short examination duration. Since DCE and DTI have similar capabilities in quantitatively assessing tumor size changes and residual tumor size (95), further large-scale studies of DTI should be performed to verify whether it can be used specifically for monitoring and evaluating the response to NAC.

#### 4.3.3 Diffusion Kurtosis Imaging (DKI)

DKI is a new MRI method to depict the diffusion of non-Gaussian water molecules in tissues. DWI is based on the assumption of the homogeneity of the microenvironment, and considers that the diffusion distribution of water molecules obeys Gaussian distribution (97). In fact, in living tissue, DWI is influenced by Brownian incoherent motion, microperfusion, and blood flow in a non-Gaussian model (98). DKI measured the tissue diffusion deviation from the Gaussian model. The ADC value corrected by the non-Gaussian distribution is called the average diffusion rate (MD). The smaller the MD value is, the more limited the diffusion motion of water molecules (94). DKI makes up for the deficiency regarding that DWI and DTI techniques cannot show the actual diffusion degree of water molecules (DKI affects the decay at high b-values). In recent years, DKI has been preliminarily applied to evaluate the efficacy of NAC in cancers [including rectal cancer (99), nasopharyngeal cancer (100), and bladder cancer (101)]. The limitation lies in the fact that the parameters are not as accurate as those of IVIM model, including the inability to distinguish between the non-Gaussian increase due to limited dispersion and multi-component confounding. Currently, there are few studies on the application of DKI parameters in the evaluation of NAC response for breast cancer. Still, preliminary results show that compared with DWI, DKI has significantly higher sensitivity and specificity in the assessment of breast cancer diagnosis and NAC efficacy (94).

### 4.4 Proton Magnetic Resonance Spectroscopy (^1^H-MRS)

The levels of choline (Cho) and its metabolites reflect the level of cellular metabolism, which is mainly involved in cell membrane transport and diffusion functions. As an active metabolite, the concentration of free Cho in normal tissues is low, and the increase in Cho level reflects an increase in cell membrane synthesis or cell proliferation. Cho peaks are significantly elevated in malignant regions, so Cho complexes are usually considered as markers of malignancy (102). ^1^H-MRS is used to assess the therapeutic effect of total choline (tCho) in malignant tumors by measuring the changes in its concentration. After effective treatment with NAC, tumor cells are damaged and their density decreases, thus the tCho peak on the MRS spectrum subsequently reduces.

In an earlier study, Jagannathan et al. (103) demonstrated that ^1^H-MRS helped in assessing the response of breast cancer to NAC. However, they used qualitative observations rather than the quantitative determination of tCho concentration to monitor tumor changes. Subsequently, several studies (104, 105) have determined the reduction in choline signal after one cycle of chemotherapy to be more sensitive than DWI-MRI in predicting pathological response. According to Bolan et al. (106), significant total choline concentration reductions were found as early as 24 hours after the initiation of chemotherapy. Furthermore, changes in tCho signal measured by MRS may provide an early indicator of treatment response than changes in size.

Due to the objective technical difficulties of ^1^H-MRS, it is currently less used. The main limitations are: 1) the low choline detection rate currently observed; 2) as the lesions shrink, less tumor tissue can be measured, especially since small lesions less than 1 cm are difficult to quantify in tCho; and 3) the relatively low sensitivity of ^1^H-MRS compared to MRI (107, 108).

**Figure 4:**
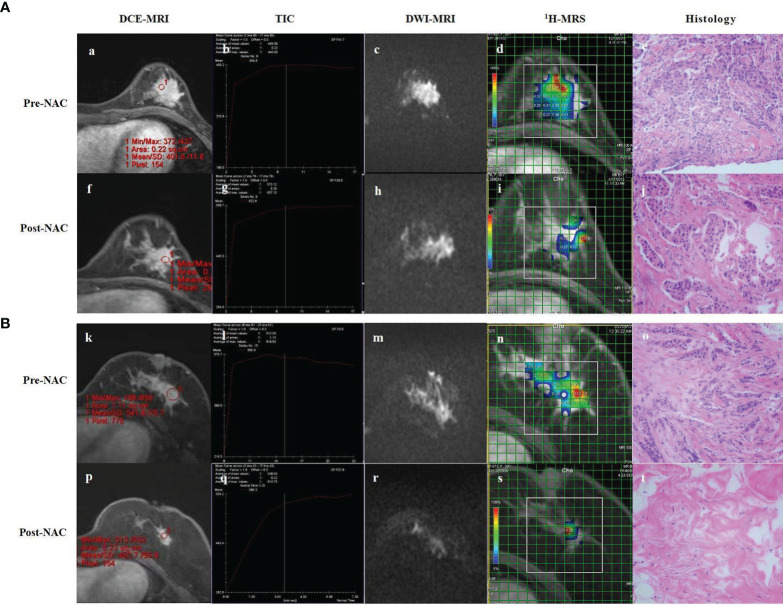
DCE-MRI, TIC, DWI-MRI, ^1^H-MRS and histology of partial responder **(A)** and complete responder **(B)** before start of NAC (Pre-NAC) and after 8 course of NAC (Post-NAC). **(A)** (a) DCE-MRI: the tumor was marked enhancement and irregular margins. (b) TIC : Fast Inflow - Platform Type. (c) DWI-MRI: the ADC value was 0.73 x 10^-3^ mm^2^/s. (d) ^1^H-MRS: high Cho levels. (e) Microscopic image of core-needle biopsy. After 8 course of NAC. (f) DCE-MRI: the mass shows a concentric shrinkage pattern. It is suggestive of partial response. (g) TIC : Fast Inflow - Platform Type. (h) DWI-MRI: the ADC value was 0.96 x 10^–3^ mm^2^/s. (i) ^1^H-MRS: lower Cho levels compared to baseline. (j) Microscopic image after NAC shows residual tumor cells, but reduced compared to baseline. **(B)** (k) DCE-MRI: the tumor was marked enhancement and irregular margins. (l) TIC : Fast Inflow - Platform Type. (m) DCE-MRI: The ADC value was 0.83 x 10^-3^ mm^2^/s. (n) ^1^H-MRS: high Cho levels. (o) Microscopic image of core-needle biopsy. After 8 course of NAC. (p) DCE-MRI: significant reduction of the mass compared to baseline. (q) TIC : Rapid Inflow - Inflow Type. (r) DCE-MRI: the ADC value was 1.39 x 10^–3^ mm^2^/s. (s) ^1^H-MRS: significant shrinkage of the tumor and a significant decrease of Cho levels. (t) Microscopic image after NAC shows lymphocyte and stromal tissue, no visible tumor cells. DCE-MRI, dynamic contrast-enhanced MRI; TIC, time-signal intensity curve; DWI-MRI¸ quantitative diffusion-weighted imaging MRI; ^1^H-MRS, ^1^H-magnetic resonance spectroscopy; NAC, neoadjuvant chemotherapy; ADC, apparent diffusion coefficient; Cho, choline.

Because of the limitations of various MRI methods and because some studies are still at the initial stages, for now, the conventional MRI, DCE-MRI, and DWI can provide more objective and comprehensive clinical information. The perfusion and diffusion MRI, which reflect the functional and molecular levels, could become important methods of imaging assessment in the future because of their quantifiable evaluation, and have also been gradually used in clinical practice (**Figure 4**). It is worth mentioning that most of the existing studies have not carefully staged breast cancer, which is why some of their results show discrepancies, especially regarding the assessment of the efficacy of the early stages of NAC. It is known that different subtypes of breast cancer respond differently to NAC; therefore, the results may be different if different proportions of patients with different molecular typing are included.

## 5 Evaluation of the Effect of PET-CT on NAC in Breast Cancer

Malignant tumors can show a high uptake of tracers because of their relatively high metabolic rate. PET-CT mainly reflects the metabolism of tissues and organs based on tracers, and reflects the changes of tumor physiological functions before and after NAC at the molecular level, which can overcome the limitations of anatomical imaging, such as MRI (109). Its effectiveness in assessing the effect of chemotherapy in breast cancer patients has been reported. Liu et al. (110) conducted a meta-analysis of six original articles (382 cases). They showed the combined sensitivity and specificity of PET-CT of 86% and 72%, respectively. Furthermore, those of MRI were 65% and 88%, respectively, suggesting that PET-CT has a higher sensitivity and lower specificity in evaluating the efficacy of NAC for breast cancer. Another meta-analysis involving 13 original studies (111) similarly compared MRI and PET-CT performance in predicting NAC efficacy, showing a combined PET-CT sensitivity and specificity of 77% and 78%, and that of MRI of 77% and 78%, respectively. This study concluded that MRI was more sensitive, and PET-CT more specific; completely contrary to the findings of previous studies. Another large sample meta-analysis that compared the performance of MRI and PET-CT in predicting the efficacy of NAC found that the timing of examination had an impact on the accuracy of both assessments. The diagnostic specificity of PET-CT was higher than that of MRI during NAC (69% vs. 42%), while the MRI sensitivity was higher after NAC (88% vs. 57%), suggesting that MRI could better assess residual tumor after treatment, while PET-CT could better assess the response during treatment (112).

The most used determination method for PET-CT is the measurement of the maximum standardized uptake value (SUVmax), which serves the purpose of early monitoring and assessment of NAC by comparing SUVmax changes before and after chemotherapy. A study added its contribution to the early screening of chemotherapy non-responders, based on a 45% decrease in SUV after the first cycle as a threshold, and a treatment non-responsive NPV of approximately 90% (113). Studies (114, 115) showed significantly correlated SUVmax of tumors with their pCR results after NAC, suggesting SUVmax as a valuable prognostic indicator. Another study (116) showed that for HER2 over-expressed breast cancer, SUVmax at the second cycle of NAC is the best indicator to evaluate efficacy. There is growing evidence that the use of PET-CT to assess metabolic response has prognostic effect on breast cancer patients treated with NAC.

PET-CT is helpful for tumor diagnosis and prognosis assessment. It has high accuracy and can be used in the early evaluation of NAC efficacy in breast cancer. Still, the specificity of PET-CT in the efficacy assessment of NAC is low; NAC is a continuous process that requires multiple tests, and the cost of PET-CT and the use of radionuclides limits its clinical application. Therefore, PET-CT has no absolute advantages over MRI. Consequently, it is not used much in clinical practice to evaluate the efficacy of NAC.

## 6 Evaluation of the Effect of NAC in Breast Cancer by Radiomics

The concept of radiomics was first proposed by one American scholar (117) in 2010 and further improved by Dutch scholar (118) in 2012. It refers to the high-throughput extraction of a large amount of information from images (CT, MRI, PET-CT, etc.) to achieve tumor segmentation, feature extraction, and model establishment; to carry out deeper mining, prediction, and analysis; and to assist imaging physicians to make the most accurate diagnosis. Currently, radiomics based on different imaging technologies such as ultrasound, mammography and MRI have been gradually applied to the differential diagnosis and prognostic analysis of breast cancer (119–121). In recent years, there have been increasing number of studies on the application of imaging omics to evaluate the efficacy of NAC for breast cancer, with several studies confirming its effectiveness. Among them, Quiaoit et al. (122) showed that imaging omics had advantages in predicting pCR after NAC for breast cancer, compared with traditional single imaging technology. Compared with single imaging assessment, imaging omics is an important emerging technology with systematic, comprehensive, and highly predictive advantages. In future, its superiority in evaluating the efficacy of NAC for breast cancer should be demonstrated.

MRI radiomics is the most commonly used technique. A recent study involving four centers (74) showed that the multi-sequence MRI model combined with T2WI, DWI, and DCE-MRI scan sequences before treatment had a higher predictive pCR ability than the single-sequence model (AUC=0.79). The predictive ability of the model for pCR in three different pathological subtypes of hormone receptor-positive, HER2 over-expressing, triple negative breast cancer, performed well in a cohort of four study centers. Another study showed a significant advantage of multivariate modeling of MRI for predicting pCR in the triple negative and HER2 positive groups before NAC (123). Radiomics combined ultrasound and PET-CT has greater potential for investigation, and recent finding showed that some radiomics features of PET and ultrasound can be considered as potential predictors of pCR (115). With the development of artificial intelligence and big data platforms, the automatic identification of breast lesions, the establishment of a multimodal intelligent and integrated diagnostic system, and the exploration of clinical mechanisms with radiomics will be gradually reflected in the clinical studies in the end.

## 7 Conclusion

In summary, various imaging methods are used to evaluate the efficacy of NAC for breast cancer in clinical practice. The value of some of the new imaging techniques has not been thoroughly studied; thus, it is not suitable for clinical application at present. With the emergence and development of new imaging techniques, we believe that certain models may exhibit high sensitivity and specificity for specific tumor subtypes. Thus, imaging evaluation is likely to become increasingly individualized. The value of mammography, ultrasound, MRI, and PET-CT in evaluating NAC in breast cancer was discussed in this paper. Additional details on the references included in this paper can be found in **Table 1**. However, breast cancer’s occurrence, development, and sensitivity to chemotherapeutic drugs are continuous, dynamic, and complex. A single imaging examination cannot provide a good evaluation of efficacy in the entire process of NAC. Therefore, in clinical treatment, we should be clearly aware of the pros and cons of various imaging methods and adopt a comprehensive method for evaluating the efficacy of NAC for breast cancer. This is expected to achieve an early, objective, and accurate assessment of efficacy, and provide a basis of decision for the precise treatment of breast cancer, ultimately improving the overall survival of breast cancer patients.

**Table 1A:** Studies on the efficacy of various imaging techniques for breast cancer NAC (References to this article) (A) Studies on the efficacy of various imaging techniques on breast cancer NAC (evaluation index: Sensitivity, Specificity).

Number	Study	Number of patients	Research type	Examination	Sensitivity (%)	Specificity (%)
1	Keune et al. (6)	192	retrospective study	US/MG	45.8/54.2	93.8/86.3
2	Skarping et al. (15)	202	prospective study	MG/US/DBT	65/62/50	81/81/91
3	Iotti et al. (22)	46	prospective study	CESM/MRI	100/87	84/60
4	Patel et al. (23)	65	prospective study	CESM/MRI	95/95	66.7/68.9
5	Barra et al. (24)	33	prospective study	CESM/MRI	76/92	87.5/75
6	ElSaid et al. (25)	21	prospective study	CESM	40	91
7	Xing et al. (26)	111	retrospective study	CESM	75-81.25	72.15-51.90
8	Amioka et al. (31)	63	prospective study	CEUS/MRI/PET-CT	95.7/69.6/100	77.5/85/52.5
9	Huang et al. (34)	143	prospective study	CEUS	78.6	74.5
10	Wang et al. (38)	290	prospective study	ABUS	85.7-88.1	81.5-85.1
11	Fernandes et al. (43)	92	prospective study	SE	84	85
12	Katyan et al. (44)	86	prospective study	SE	97.7-77.8	68.7-100
13	Jing et al. (45)	62	prospective study	SWE	72.92	85.71
14	Lee et al. (46)	71	prospective study	US/SWE	72.1/83.6	50/80
15	Maier et al. (48)	134	prospective study	SWE	79.6	58.6
16	Sannachi et al. (49)	30	prospective study	QUS	82	100
17	Yu et al. (52)	20	prospective study	OPTI-MUS	76.9	71.4-85.7
18	Altoe et al. (54)	40	prospective study	OPTI-MUS	86.7	68.4
19	Tran et al. (55)	22	prospective study	QUS+OPTI-MUS	64.3-100	62.5-100
20	Bouzon et al. (58)	91	prospective study	MRI	75	78.57
21	Cheng et al. (66)	969	meta-analysis	DCE-MRI	80	84
22	Zheng et al. (70)	63	prospective study	DCE-MRI	66.8-75.0	60.0-66.7
23	Fukuda et al. (72)	265	prospective study	DCE-MRI	43.2	97.7
24	Zhu et al. (78)	64	prospective study	DWI-MRI	91.67	87.5
25	Richard et al. (79)	118	retrospective study	DWI-MRI	100	38
26	Liu et al. (81)	176	retrospective study	DWI-MRI	62.5-75	82.61-97.36
27	Che et al. (87)	36	prospective study	IVIM-MRI	100	73.7
28	Jagannathan et al. (103)	67	prospective study	^1^H-MRS	78	86
29	Tozaki et al. (104)	34	prospective study	^1^H-MRS	/	/
30	Bayoumi et al. (108)	47	prospective study	^1^H-MRS+DCE-MRI	75	97.1
31	Liu et al. (110)	382	meta-analysis	(18)F-PETCT/MRI	86/65	72/88
32	Li et al. (111)	1193	meta-analysis	MRI/PETCT	0.88/0.77	0.69/0.78
33	Sheikhbahaei et al. (112)	595	meta-analysis	MRI/PETCT	0.88/0.71	0.55/0.77
34	Schwarz-Dose et al. (113)	87	prospective study	PETCT	69-73	63
35	Akimoto et al. (114)	130	prospective study	(18)F-PET/CT	79.3	53.1

**Table 1B:** Studies on the efficacy of various imaging techniques for breast cancer NAC (References to this article) (B) Studies on the efficacy of various imaging techniques on breast cancer NAC [evaluation index: correlation coefficient (CC)].

Number	Study	Number of patients	Research type	Examination	CC
1	Leddy et al. (14)	57	prospective study	Ultrasonic/MM/MRI	0.71/0.58/0.50
2	Kim et al. (16)	207	prospective study	MG/MRI	0.368/0.823
3	Um et al. (17)	151	prospective study	MRI/MG	0.769/0.651
4	Fallenberg et al. (20)	178	prospective study	MG/CESM/MRI	0.61/0.69/0.79
5	Cao et al. (32)	31	prospective study	CEUS	0.976
6	Lee et al. (33)	30	prospective study	CESM/MRI	0.75/0.42
7	Park et al. (40)	51	prospective study	MG/DBT/ABUS/MRI	0.56/0.63/0.55/0.83
8	Segara et al. (63)	68	retrospective study	MRI/US/physical exam	0.869/0.612/0.439
9	Newitt et al. (64)	20	prospective study	DWI-MRI	0.91-0.92
10	Furman-Haran et al. (96)	20	retrospective study	DTI-MRI	0.82
11	Tozaki et al. (105)	9	prospective study	^1^H-MRS	0.91
12	Antunovic et al. (115)	79	retrospective study	(18)F-PET/CT radiomics	0.7-0.73
13	Zhuang et al. (122)	144	retrospective study	MRI radiomics	0.826-0.902

**Table 1C:** Studies on the efficacy of various imaging techniques for breast cancer NAC (References to this article) (C) Studies on the efficacy of various imaging techniques on NAC in breast cancer (evaluation index: AUC).

Number	Study	Number of patients	Research type	Examination	AUC
1	Dromain et al. (21)	110	prospective study	MX ± US ± CEDM/MX ± US	0.87/0.83
2	Xing et al. (26)	111	retrospective study	CESM	0.733-0.776
3	Lee et al. (46)	71	prospective study	US+SWE/MRI	0.877/0.939
4	Evans et al. (47)	80	prospective study	US+SWE/MRI	0.92/0.96
5	Rauch et al. (51)	33	prospective study	OPTI-MUS	0.92
6	Zheng et al. (70)	63	prospective study	DCE-MRI	0.703-0.767
7	Loo et al. (71)	54	prospective study	DCE-MRI	0.73
8	Liu et al. (74)	586	retrospective study	MRI radiomics	0.86
9	Galban et al. (75)	39	prospective study	DWI-MRI	0.825
10	Minarikova et al. (76)	42	prospective study	DWI-MRI	0.79
11	Iwasa et al. (77)	24	prospective study	DWI-MRI	0.9
12	Bufi et al. (80)	225	retrospective study	DWI-MRI	0.587
13	Liu et al. (81)	176	retrospective study	DWI-MRI	0.751-0.864
14	Xu et al. (88)	51	prospective study	IVIM-MRI	0.832
15	Wilmes et al. (95)	34	prospective study	DTI-MRI	0.6-0.83
16	Bolan et al. (106)	119	prospective study	^1^H-MRS	0.51-0.53
17	Li et al. (111)	1193	meta-analysis	MRI/PETCT	0.88/0.84
18	Luo et al. (119)	315	prospective study	US radiomics	0.928
19	Quiaoit et al. (121)	36	prospective study	US radiomics	0.87
20	Cain et al. (123)	288	prospective study	DCE-MRI radiomics	0.707

## Author Contributions

XK and QZ equally contributed to the manuscript. XK and QZ reviewed the literature, and wrote the manuscript. XW, TZ, JD, SS and JN contributed to developing the manuscript,and drafting and revising the text, tables, and figure. CT, MT, MW, JZ and YX revised the manuscript. ZHL and ZL designed and revised the manuscript.

## Funding

This study was supported by grants from the National Nature Science Foundation of China (No.82060481); the Outstanding Youth Science Foundation of Yunnan Basic Research Project (202101AW070001); the Yunnan Applied Basic Research Projects (No.202001AT070046); the Yunnan Province technology innovation talent training object project (No. 202105AD160014); and the Graduate Innovation Fund project of Kunming Medical University (No. 2021S252).

## Conflict of Interest

The authors declare that the research was conducted in the absence of any commercial or financial relationships that could be construed as a potential conflict of interest.

## Publisher’s Note

All claims expressed in this article are solely those of the authors and do not necessarily represent those of their affiliated organizations, or those of the publisher, the editors and the reviewers. Any product that may be evaluated in this article, or claim that may be made by its manufacturer, is not guaranteed or endorsed by the publisher.

## Abbreviations

NAC, neoadjuvant chemotherapy; pCR, pathological complete responses; US, ultrasound; MRI, magnetic resonance imaging; PET-CT, positron emission tomography CT; CR, complete remission; PR, partial response; PD, disease progression; SD, stable disease; CCC, consistency correlation coefficient; PPV, positive predictive value; NPV, negative predictive value; CESM, contrast-enhanced spectral mammography; ΔCGV, grey value reduction percentages; CEUS, contrast-enhanced ultrasound; ABVS, automated breast volume scanner; DBT, digital breast tomosynthesis; SE, Strain elastography; SWE, shear wave elastography; AUC, area under curve; QUS, quantitative ultrasound; OPTI-MUS, diffused optical tomography with ultrasound; RF, radio frequency; FFT, fast Fourier transform; SS, spectral slope; SI, spectral intercept; MBF, mid-band fit; ASD, average scatterer diameter; AAC, average acoustic concentration; ACE, attenuation coefficient estimate; SAS, spacing among scatterers; HBT, total hemoglobin; HBO2, deoxyhemoglobin; DCE-MRI, dynamic contrast-enhanced MRI; TIC, time-signal intensity curve; DCIS, ductal carcinoma in situ; DWI-MRI¸ quantitative diffusion-weighted imaging MRI; ROC, receiver operating characteristic; CI, confidence interval; ADC, apparent diffusion coefficient; IVIM, quantitative Intravoxel incoherent motion; ^1^H-MRS¸^1^H-magnetic resonance spectroscopy; tCho, total choline; DTI, diffusion tensor imaging; DKI, diffusion kurtosis imaging.
